# Iterative transfer learning for automatic collective motion tuning on multiple robot platforms

**DOI:** 10.3389/fnbot.2023.1113991

**Published:** 2023-03-16

**Authors:** Shadi Abpeikar, Kathryn Kasmarik, Matt Garratt

**Affiliations:** School of Engineering and IT, University of New South Wales, Canberra, ACT, Australia

**Keywords:** swarm, robot, reinforcement learning, transfer learning, coverage

## Abstract

This paper proposes an iterative transfer learning approach to achieve swarming collective motion in groups of mobile robots. By applying transfer learning, a deep learner capable of recognizing swarming collective motion can use its knowledge to tune stable collective motion behaviors across multiple robot platforms. The transfer learner requires only a small set of initial training data from each robot platform, and this data can be collected from random movements. The transfer learner then progressively updates its own knowledge base with an iterative approach. This transfer learning eliminates the cost of extensive training data collection and the risk of trial-and-error learning on robot hardware. We test this approach on two robot platforms: simulated Pioneer 3DX robots and real Sphero BOLT robots. The transfer learning approach enables both platforms to automatically tune stable collective behaviors. Using the knowledge-base library the tuning procedure is fast and accurate. We demonstrate that these tuned behaviors can be used for typical multi-robot tasks such as coverage, even though they are not specifically designed for coverage tasks.

## 1. Introduction

Applying deep learning algorithms, including deep reinforcement learning, to solve complex problems requires a significant effort in data collection to inform the design of components such as the reward signal (Sutton and Barto, 2018). Collective motion behavior tuning for robots using deep learning is one application that suffers from this challenge (Schranz et al., 2020). Collecting large training datasets for robots is both time-consuming and can result in wear and tear or damage to the robots. This paper aims to address this challenge by proposing a methodology for collective motion behavior tuning that requires only a very small set of data to be collected up front from robots.

Swarming collective motion is inspired by flocks of birds, herds of land animals, schools of fish, and swarms of insects. This behavior enables them to do their tasks efficiently, and collectively. Therefore, it can also be an efficient motion for robots (Savkin, 2004; Qadri et al., 2021), called swarm robots (Navarro and Mat́ıa, 2013; Schranz et al., 2020). Some instances of swarm robots' missions include exploration (Huang et al., 2019), path formation (Sperati et al., 2011), and self-organized aggregation (Khaldi et al., 2018). However, the key difficulty with the existing works is that swarm parameters must be manually tuned. This will raise the cost and time required for hand-tuning the behaviors, as well as the inaccuracy.

To automatically generate collective behavior for robots, Toshiyuki et al. (2016) developed a self-organized flocking behavior for leader and follower movement of two-wheeled robots. Tran et al. (2022) provided a frontier-led swarming behavior for multi-robot coverage problems. Firat et al. (2020) proposed self-organized aggregation using informed robots. Although these works provide automatic collective behaviors generation, none of them provides a diverse set of collective behaviors, applicable to different robot platforms.

Recent works proposed automatically tuning more diverse collective behaviors. Khan et al. (2020) proposed an evolutionary approach, which requires a large training time to extract data for the evolved diverse collective behaviors. Abpeikar et al. (2022a) introduced a reinforcement learning (RL) algorithm for automatic collective motion tuning (called CoMoT) in simulated point mass boids. This system is able to automatically provide a diverse set of behaviors because an extensive human study provided data labels for “swarming” and “not swarming” behaviors (Abpeikar and Kasmarik, 2019; Abpeikar, 2022). As the main challenge collecting this labeled data took 6 months. Moreover, this system could only address point mass boids and is not applicable to real robots. On the other hand, designing such a system for real robots is still challenging, since in real robots, collecting training data is very costly as humans would need to watch multiple robot behaviors to label data. Moreover, fine-tuning the reinforcement learning parameters and the trial-and-error procedure of reinforcement learning will increase this cost. It also might result in damage to the robots when the algorithm is not yet well-trained and collisions still occur.

This paper aims to develop an automatic collective motion tuner for real robots the same as CoMoT, with no need for huge data collection from robots. This approach will eliminate the challenges required for collecting huge training data for robots since it is transferable between point-mass boids and different robot platforms. Moreover, there is no need for designing reinforcement learning from scratch and applying the trial-and-error procedure of RL on real robots. Eliminating these needs will result in saving time and cost. Hence, this paper developed a system that can leverage the available human-labeled swarming collective motion data to recognize and tune swarming collective motion across multiple robot platforms. This can be done by generating a library for transfer between point-mass and robots, using a transfer learning applicable to the observation space of CoMoT reinforcement learning. The library is able to use a small set of robot data and could transfer this data to the available huge human-labeled data. Regarding this, the contributions of this paper are as follows:

- To reduce the risks and time required for the data collection from real robots and the trial-and-error procedure of reinforcement learning, this paper applies transfer learning on CoMoT to automatically tune collective behavior for robots. The novelty is to propose an algorithm that applies transfer learning (Zhong et al., 2018) to the observation (state) space of a CoMoT reinforcement learning agent. Consequently, the observation space of robots could be matched with that of the boids. Using transfer learning, tuning collective behavior for robots will be off-line. Hence, CoMoT can tune collective behavior for robots rather than simulated point masses, while never being trained on real robots.
- An iterative library update algorithm for transfer of learning between boids and robot platforms. This iterative approach requires only a small amount of initial data from each robot platform, which can be collected from random movements. Using this iterative procedure, the library will be continuously extended. Consequently, there is no need to hand-tune collective behaviors to enrich the training set, since the random set is enough for the iterative library update. The extracted library could speed up the collective motion tuning for robots to occur in <10 s.
- Using transfer learning, a CoMoT agent which is trained with point-mass boid data could automatically tune collective behavior for different robot platforms. The only requirement is that the robot platforms have shared parameters with boids, no matter if their characteristics including size, speed, and shape are different. An evaluation of our approach on two different robot platforms: Pioneer 3DX and Sphero BOLT, shows that transfer learning on these real robots is fast and accurate.
- Although the CoMoT reinforcement learning agents has never been trained for a specific mission, a demonstration has been presented which shows that tuned behaviors can be applied to real-world problems such as area coverage problems.

The remainder of this paper is organized that Section 2 discusses the related works to develop RL for collective motion tuning in robots. Section 3 illustrates the iterative transfer learning on the observation space of RL. Section 4 includes the experimental analysis of the proposed method. Finally, Section 5 concludes the paper by indicating some ideas for future works.

## 2. Background and related work

This section includes related work relevant to automatic tuning of collective motion. Section 2.1 provides a brief definition of collective motion behavior. Section 2.2 discusses recent work developing collective behavior using RL. Section 2.3 indicates existing transfer learning methods applied on RL. Section 2.4 illustrates the characteristics of Sphero BOLT and Pioneer 3DX robots used in this paper.

### 2.1. Collective motion behavior

Collective motion behavior is inspired by flocks of birds and schools of fish, and the way they move collectively. The first computer-based collective behavior was introduced by Reynolds (Reynolds, 1987). This model is known as Reynolds' boid model. The Boid Guidance Algorithms (BGAs) use three simple rules of cohesion (stay close together), separation (do not run into each other), and alignment (move in the same direction) of Reynolds' boid model to provide collective behavior in boids and robots (Khan et al., 2020). The Boid Guidance Algorithms apply swarming collective motion parameters of cohesion ($C_{t}^{i}=\left(C_{x_{t}}^{i},C_{y_{t}}^{i}\right)$), separation ($S_{t}^{i}=\left(S_{x_{t}}^{i},S_{y_{t}}^{i}\right)$), alignment ($A_{t}^{i}=\left(A_{x_{t}}^{i},A_{y_{t}}^{i}\right)$), and the corresponding weights (*W*_*c*_, *W*_*s*_, *W*_*a*_) to control these three rules. The probability forces of *P*_*s*_, *P*_*c*_, and *P*_*a*_ are used in the Boid Guidance Algorithm by Khan et al. (2020) to control the three corresponding rules of Reynolds' boid model. Hence they could control the formation of the collective motion behaviors. Each one refers to a probability value in range of [0, 1]. These probabilities indicate the likelihood that each corresponding rule will be applied on each boid at each timestep of the motion (Khan et al., 2020). They control the frequencies of the separation, cohesion, and alignment updates and consequently the frequencies of velocity and position updates of the boids in each timestep. Assigning different values to these parameters will result in different motion formations for boids (Khan et al., 2020). Using these parameters on a group of boids within predefined values for cohesion, separation, and alignment radii (*R*_*c*_, *R*_*s*_, *R*_*a*_) are used to update the velocity of boids $V_{t}^{i}=\left(V_{x_{t}}^{i},V_{y_{t}}^{i}\right)$, using Equation (1) (Khan et al., 2020). In this equation, *t* refers to the simulation timestep, and *i* refers to boid index in the predefined corresponding radii. Also, the velocity vector $V_{t}^{i}=\left(V_{x_{t}}^{i},V_{y_{t}}^{i}\right)$ is computed based on the distance moved in pixels by boid *i* at each tick of the simulation timestep ($\frac{pixel}{tick}$).


(1) $$\begin{array}{l} V_{t+1}^{i}=V_{t}^{i}+W_{c}C_{t}^{i}+W_{s}S_{t}^{i}+W_{a}A_{t}^{i} \end{array}$$

The updated velocity vector $V_{t}^{i}=\left(V_{x_{t}}^{i},V_{y_{t}}^{i}\right)$ then will change the boids' position (*P* = (*x, y*)), using Equation (2).


(2) $$\begin{array}{l} P_{t+1}^{i}=P_{t}^{i}+V_{t+1}^{i} \end{array}$$

These parameters are correlated with each other based on the Boid Guidance Algorithm proposed by Khan et al. (2020) and the velocity and position updates by Equations (1), (2). These three rules, and the velocity and positioning update, will provide a collective behavior motion for boids. The most effective values to generate a collective behavior is recognized by Khan et al. (2020) and investigated in a human study by Abpeikar et al. (2022a). The boids model has been extended to provide a rules base for guiding swarms of robots and is a simple yet effective way to efficiently guide the paths of multiple robots working together (Trianni, 2008). This is behavior is called swarm robotics in the literature (Savkin, 2004). An effective swarm robotics algorithm needs to make robots stay close and connected, move in the same direction, and without running into each other. In many existing methods, humans hand-tune the swarming collective motion parameters to generate collective behavior for robots, which increases the risk of human involvement. This paper aims to automatically tune these swarming collective motion parameters to generate collective behavior for robot platforms, using the transfer learning approach. For more information on collective behavior see e.g., Reynolds (1987).

### 2.2. Collective behavior by reinforcement learning

Developing collective behavior automatically for a group of robots is challenging (Francesca and Birattari, 2016). Although there are some existing works which simulate swarming collective behavior in robots, including collective navigation for robots (Na et al., 2022), collaborative robots (Aydin and Fellows, 2018), and collective formation of robots (Buffet et al., 2007), none of these can automatically generate a diverse set of collective behaviors. One limitation in doing this is that automatic recognition of swarming collective motion behavior is hard (Harvey et al., 2018). It is hard for a machine, however, humans can recognize this behavior easily. Hence, recent works used human perception to train machines for automatic collective behavior recognition in simulation (Kasmarik et al., 2020; Abpeikar et al., 2022b). Following this approach, some success has been achieved for automatic collective motion generation with RL. RL is a trial-and-error method, which can solve complex problems. It takes an observed state of the environment as input in each iteration, then selects an action from the action space, which maximizes the corresponding reward (Sutton and Barto, 2018). Using human-labeled data as a knowledge base for reward signal generation enables RL to do automatic collective motion tuning (CoMoT) in simulated robots (Abpeikar et al., 2022a), and collective motion tuning for environmental sensing (Abpeikar et al., 2022c). However, these methods are only applicable on simulated robots. This paper extends CoMoT (Abpeikar et al., 2022a) to automatically recognize and tune collective motion on robot platforms rather than point-masses.

### 2.3. Transfer learning on reinforcement learning

The idea of transfer learning is to train the learner on a source problem in which a large training set is accessible and transfer the trained knowledge to a target problem (Torrey and Shavlik, 2010). The target problem must have some common characteristics with the source problem, but the amount of training data may be smaller (Torrey and Shavlik, 2010). Since RL is able to solve complex problems, applying transfer learning to RL has many advantages including reducing the costs of training from scratch (Feuz and Cook, 2015). Transfer learning on RL includes three types (Zhong et al., 2018): instance-based, feature-based, and parameter-based. Since collective behavior data of simulated boids, simulated robots and real robots, have the same set of features, but with different observation space ranges and distributions (Abpeikar et al., 2022b), the proposed method in this paper focuses on feature-based (observation space) transfer learning. Some feature-based transfer learning methods applied to RL are based on distribution similarity (Zhong et al., 2018), model-based regularization (Sun et al., 2022), and feature-space re-mapping (Feuz and Cook, 2015). The transfer learning on observation space used in this paper is based on using the Kullback-Leibler Divergence (KLD) method described by Zhong et al. (2018). KLD is a well-known approach to finding distribution differences between two feature sets (Zhong et al., 2018). This paper uses KLD to find distribution differences between the observation space of boids and the observation space of a chosen robot platform, and map these spaces into each other. The KLD approach will result in a library for transferring learning between boids and the chosen robot platform. A detailed discussion on how KLD will be used in the observation space of CoMoT will be given in Section 3.1.

### 2.4. Robot platforms

This paper considers two robots platforms: Real Sphero BOLT, and Simulated Pioneer 3DX Robots.

**Figure 1:**
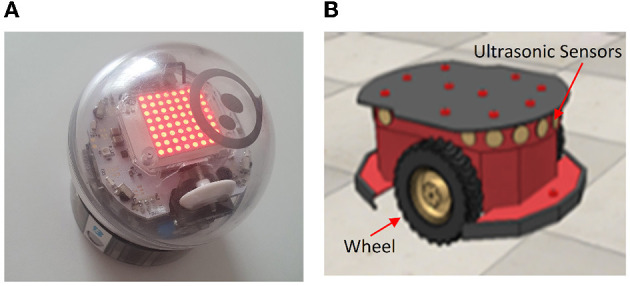
Sphero BOLT and Pioneer 3DX **(A, B)**—the two robot platforms used in the experiments of this paper.

The Sphero BOLT is a commercially available robot, that can be programmed using Python. Sphero BOLT is a small, lightweight, 2-wheeled differential drive robot. It is fast, with a top speed of 2.25*ms*^−1^. Multiple robots can be controlled from a single laptop so it's possible to transport all the necessary equipment for a swarm in a small suitcase. As shown in Figure 1A, the shell of the Sphero BOLT is a clear plastic, which is waterproof and durable and it has a long-lasting battery. Communication with the Sphero BOLT robot is *via* Bluetooth enabling it to be controlled and monitored from compatible devices such as smartphones and laptops. The sensors of the Sphero BOLT include a light sensor, gyroscopes, accelerometers. Also, it has a separate drive motor for each wheel.^1^ This paper runs an experiment with three Sphero BOLT robots in a 2 × 2*m* environment. The environment is a virtual walled environment, and the Sphero BOLT robots are programmed to reflect these virtual walls. The motor encoder is used for the positioning. Moreover, the magnetometer and motor encoders used together for the velocity computation. The initial hand-crafted movements will be discussed in detail in Section 4.1.

The Pioneer 3DX robot (Figure 1B) is a larger 2-wheeled differential drive robot, equipped with 16 ultrasonic sensors. The wheels and the 16 ultrasonic sensors are presented in Figure 1B. The top speed of the Pioneer 3DX robot is 2 *ms*^−1^. Robots can again be controlled from a laptop (over a WiFi network), but as these robots are heavier a small van is needed to transport a swarm of multiple robots. For the work described in this paper, we run simulations with eight Pioneer 3DX robots in a 20 × 20*m* environment implemented in the CoppeliaSim simulator. The CoppeliaSim is used to collect the position and velocity data. Moreover, a weight has been defined in the simulator to implement the wall reflection. More details on the different simulated scenarios are provided in Section 4.1. For more information on Pioneer 3DX robots, see e.g., Kasmarik et al. (2020).

## 3. Methodology: Collective motion tuning in robots using transfer learning

In this section, the methodology for designing an automatic system for collective motion tuning in robots using reinforcement learning trained on boids is articulated. To transfer the knowledge from boids to robots and automatically tune this behavior, four procedures are required: (1) Recognition of collective motion; (2) Tuning collective motion for boids by RL; (3) Iterative transfer learning on the observation space of the RL; and (4) automatic collective motion tuning for robots. These procedures will be described in detail in the following sections. Procedures (1) and (2) are from existing work, while procedures (3) and (4) form the contribution of this paper. The contribution of this paper–iterative transfer learning to extend CoMoT to real robots–is discussed in Sections 3.3, 3.4.

### 3.1. Collective motion recognition

To exploit the human ability to recognize collective behavior recognition, one recent approach collected data from humans regarding their opinion of different behaviors. Then this labeled data was used to train a machine to mimic human recognition of swarming collective motion (Kasmarik et al., 2020). Human labels were collected *via* an online survey.^2^ The collected dataset includes 4,803,200 samples with 12 features. The survey was used to construct a binary dataset of labeled structured and unstructured collective motions (Abpeikar, 2022). “Structured” referred to a motion with an embedded pattern. Various machine learning algorithms were tried to see which could best mimic human recognition using this dataset as a training set. Following these experiments, the decision tree resulted in the maximum accuracy (Kasmarik et al., 2020). A knowledge base of 73 if-then rules was extracted from a pruned version of this decision tree (Abpeikar et al., 2022a). The if-then rules of this knowledge demonstrated fast and accurate swarming collective motion recognition of boids and some simulated robots (Abpeikar et al., 2022b). This knowledge base was then used as a reward signal generator inside CoMoT. The RL component of CoMoT will be discussed in Section 3.2.

### 3.2. Reinforcement learning for collective motion tuning of boids

**Figure 2:**
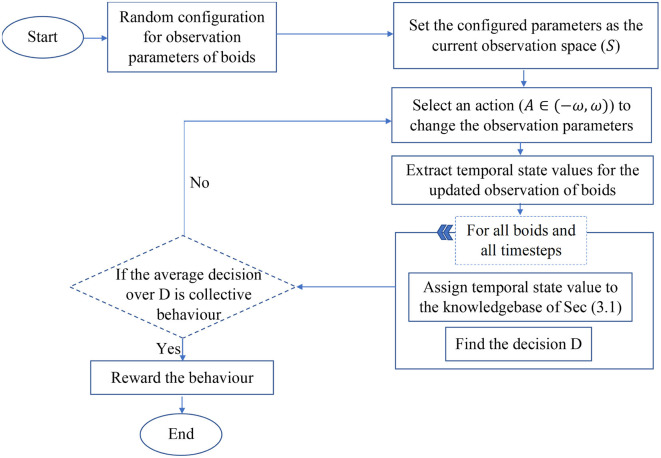
Flowchart of the CoMoT learning procedure.

RL can be used to tune collective motion by allowing an agent (such as CoMoT Abpeikar et al., 2022a) to observe a group of agents moving and perturb their movement by changing the parameters of their behavior. CoMoT uses the knowledge base described in Section 3.1 as the reward signal generator. Each episode of CoMoT follows the procedure presented in Figure 2.

The observation space of CoMoT comprises features for boids' collective motion parameters, including, maximum and minimum speed (*V*_*max*_, *V*_*min*_), separation, alignment, and cohesion weights (*W*_*s*_, *W*_*a*_, *W*_*c*_) and their corresponding radius (*R*_*s*_, *R*_*c*_, *R*_*a*_) and probability forces (*P*_*s*_, *P*_*c*_, *P*_*a*_) as *S* = (*W*_*s*_, *W*_*a*_, *W*_*c*_, *V*_*max*_, *V*_*min*_, *R*_*s*_, *R*_*c*_, *R*_*a*_, *P*_*s*_, *P*_*c*_, *P*_*a*_). The action space includes perturbations in the range of these parameters, which makes small changes to the collective motion parameters.Based on the discussion provided in Abpeikar et al. (2022a), *ω* = [0.5, 0.5, 0.5, 0.1, 0.1, 10, 50, 50, 0.1, 0.1, 0.1] is the small perturbation value which used as an action to change each parameter of the current state [*W*_*s*_, *W*_*a*_, *W*_*c*_, *V*_*max*_, *V*_*min*_, *R*_*s*_, *R*_*c*_, *R*_*a*_, *P*_*s*_, *P*_*c*_, *P*_*a*_], respectively. Therefore, the selected action should be in the range *A* ∈ [−*ω*, *ω*]. Then the temporal state values of boids is collected for 10 timesteps while they are moving in a rectangular area. The temporal state values include *x* and *y* coordinates of velocity (*V* = (*V*_*x*_, *V*_*y*_)), separation (*S* = (*S*_*x*_, *S*_*y*_)), alignment (*A* = (*A*_*x*_, *A*_*y*_)) and cohesion (*C* = (*C*_*x*_, *C*_*y*_)) in each timestep for each boid. Then the temporal state values are fed to the knowledge-base of the reward signal. The knowledge-base rewards temporal state values that are recognized as collective behavior and returns a negative value for behaviors recognized as random. The reward signal generates an average over the 10 temporal state rewards as the final decision. Then an actor-critic network learns the policy for maximizing the future rewards.

CoMoT requires 2,000 episodes, each with a maximum of 100 steps before it can accurately tune a swarming collective behavior. In addition to this cost, there is a substantial overhead of generating the training dataset from an online survey (described in Section 3.1), not to mention the costs of designing the reward signal, rate of perturbation of the action space, and the architecture of the actor-critic network (Abpeikar et al., 2022a). The idea of incorporating transfer learning in CoMoT aims to make it possible to use the system on real robots without incurring these costs again.

### 3.3. Transfer learning for collective motion tuning in robots

Although the observation space parameters of point mass boids and robots' are the same when they are engaged in collective behavior, the ranges and distributions of the parameter values are different. This is because robots have a volume, wheelbase, wheel diameter, top speed and so on that makes their movement properties different to point masses. These differences raise the challenges of: (1) training from scratch for collective motion tuning on each robot platform, and (2) the need for collecting a large amount of training data. Designing from scratch would require the effort of fine-tuning the parameters mentioned in Section 3.2. On the other hand, transfer learning can deal with these issues by gathering a small training dataset from the robots–just enough to determine the difference in the distribution of parameter value ranges. Then the transferred observation space can be used for automatic swarming collective motion generation in robots. Section 3.3.1 investigates the methodology for determining the differences in the distribution of parameters. Section 3.3.2 illustrates the transfer learning approach on the observation space of CoMoT.

#### 3.3.1. Extended kullback-leibler divergence for transfer learning on observation space

As mentioned in Section 2.2, there are different types of transfer learning. Since the major difference between collective motion tuning for boids and robots are the ranges of parameters in observation space, the best type of transfer learning to use is the feature-based approach. As a feature-based approach in this paper, we will investigate to make the distribution of target data the same as the source data. One popular method to identify the distribution of the feature space is Kullback-Leibler Divergence (KLD) (Zhong et al., 2018). KLD looks for similar features in each missing dataset and finds the distribution related to that feature. We propose Algorithm 1 based on KLD, as the method to transfer learning in the observation space of RL. This algorithm can be used for two purposes: (1) to transfer from boid data to robot data and (2) to transfer back from robot data to boid data. This facilitates the full feedback loop required for behavior tuning. In the transfer from boid data to robot data, the “source data” refers to the boid data, while the “target data” refers to the robot data. This definition is the opposite, when transferring from robot data to boid data. The proposed transfer learning algorithm has the following difference compared to Zhong et al. (2018):

- It works on all features of target data, and not only the missing data. The idea is to make the distribution for all features of the target data similar to those of the source data. This is a requirement since any change in the observation space makes the RL inappropriate.
- The update procedure (Equation 4 and Line 3 of Algorithm 1) in the proposed transfer learning not only provides the same distribution, but also imparts the same space ranges.
- The data feature in Algorithm 1 refers to both configuration parameters (*Data*_*conf*_) and the temporal state parameters (*Data*_*temp*_). However, the work of Zhong et al. (2018) could only work with a set of temporal data.
- The transfer learning could work from boid data to robot data and conversely. However, the work of Zhong et al. (2018) could only work with the richer data as the source data.

**Algorithm 1:** Transfer from Source to Target data

1 Compute *PD*_*S*_*j*__ for all *j* features in source data, and *PD*_*T*_*k*__ for all *k* features in the target data.	2 For each feature *k* in the target data	2.1 For each feature *j* in source data	Compute the *KLD*_*j*_	2.2 Find the feature *S*′ ∈ *j* in source data which has maximum *KLD*_*j*_.	2.3 Return *S*′ as the similar feature to *k* in source data	3 For each sample *i* and feature *k* in target data	3.1 Use the similar feature *S*′ of source data.	3.2 Update target sample, using Equation (3)	4 Return:	4.1 *S*′ for each feature *k* of the target data.	4.2 The Updated Target Samples (UPS).	4.3 Mean values of each feature of source and target data.

In Algorithm 1, the first step finds common features between source and target data, with respect to each feature of the target data. Then, each sample of target data is updated using the corresponding similar feature in the source data, using Equation (3). In this equation, *T* refers to the target, *S* refers to the source, (*i, j*) refers to sample *i* and feature *j*. Also, ${\bar{S}}_{S^{\prime }}$ is the mean of all samples of the selected similar feature of the source data and ${\bar{T}}_{j}$ is the mean of all samples of feature *j* in the target data. *S*′ is a similar feature of source data, corresponding to feature *j* in the target data. Dividing ${\bar{S}}_{S^{\prime }}$ (the mean of source samples) by ${\bar{T}}_{j}$ (the mean of target samples), will map the target samples more closely to the mean of source samples, and reduces the centrality of the mean of the target samples. This will shift the distribution probability of target samples to the source samples. Therefore, the updated target samples will have a distribution probability with the mean value closer to the source mean value.


(3) $$\begin{array}{l} transfer\left(S_{T}\left(i,j\right)\right)=S_{T}\left(i,j\right)\times \left({\bar{S}}_{S^{\prime }}÷{\bar{T}}_{j}\right) \end{array}$$

Similar features could be found using the KLD metric. The KLD metric is computed using Equation 4, Zhong et al. (2018). In this equation, *PD*_*S*_*j*__ is the probability distribution of all samples of feature *j* of the source data. Also, *PD*_*T*_*k*__ is the probability distribution of all samples of all features *k* = 1, ..., *k*′ of the target data. *k*′ is the total number of features in the target data.

While Algorithm 1 can provide transfer from boid data to robot data and inversely, the procedure of Algorithm 1 requires 10 min to find the transfer learning parameters for real-time problems. Therefore, to eliminate the need for running Algorithm 1 for each tuning step by CoMoT, Section 3.3.2 will propose the transfer learning, and Section 3.4 proposes a framework to create a library for transferring the data without requiring any further training.


(4) $$\begin{array}{l} KLD_{j}=\underset{k}{\sum }\left(PD_{S_{j}}\times \log\left(PD_{S_{j}}/PD_{T_{k}}\right)\right) \end{array}$$

#### 3.3.2. Transfer learning on observation space of CoMoT

As has been discussed in the previous section, Algorithm 1 by using KLD is able to transfer the data distribution of boids to robots and conversely, depending on the data selected as source data. To deal with the slow speed of Algorithm 1, we will propose a library extraction in this section. Two approaches for library extraction are proposed in this paper, which will be discussed in detail in the following sections.

##### 3.3.2.1. One shot transfer

**Figure 3:**
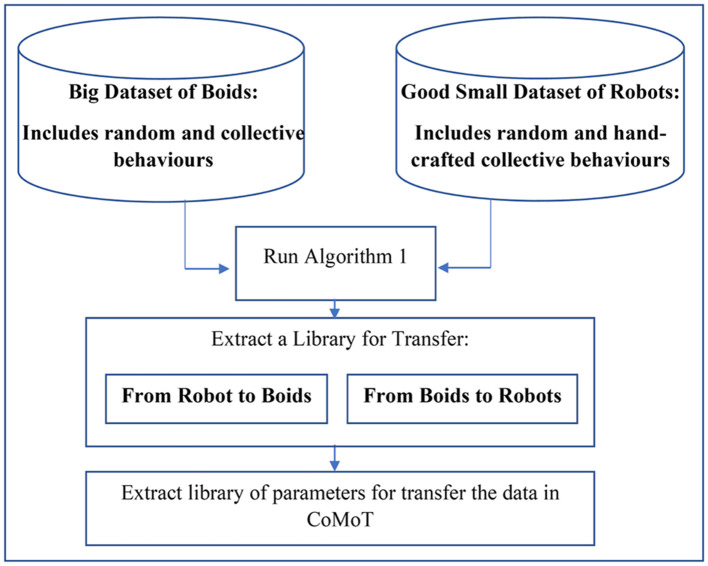
One shot transfer learning to extract a library for CoMoT.

This approach uses a traditional transfer learning approach. It uses a small set of hand-crafted behaviors in robots and generates two libraries to transfer from boids to robots and conversely. This method is presented in Figure 3. As shown in this figure, this library uses some simple training samples from robots and a huge training dataset from boids. The collective and random behaviors of robots introduced to the library are a set of hand-crafted behaviors. To generate a library for transfer from boids to robots and conversely, Algorithm 1 is applied twice by swapping the source and target data. Then the parameters of Algorithm 1 for transfer from the source to the target data will be extracted. These parameters are $\bar{S_{S^{\prime }}}$, $\bar{T_{j}}$, and the similar feature of source data, corresponding to each feature *j* of the target data, described in Section 3.3.1. Using the library of these parameters and Equation 3 on each target data record, CoMoT can transfer the observation space of the robots to the boid data. This transfer enables CoMoT to tune a collective behavior for robots by pretending they are boids.

Although using this approach will facilitate the automatic swarming collective motion generation of robots, the downside is that we had to first hand-craft collective behaviors in the initial stage of Figure 3. Hand-crafting collective behaviors for robots needs time and effort and somewhat undermines the purpose of our mission to generate them automatically. To overcome this issue, the following section will introduce an iterative approach, starting from a weaker, smaller random behavior dataset.

##### 3.3.2.2. Iterative transfer

**Figure 4:**
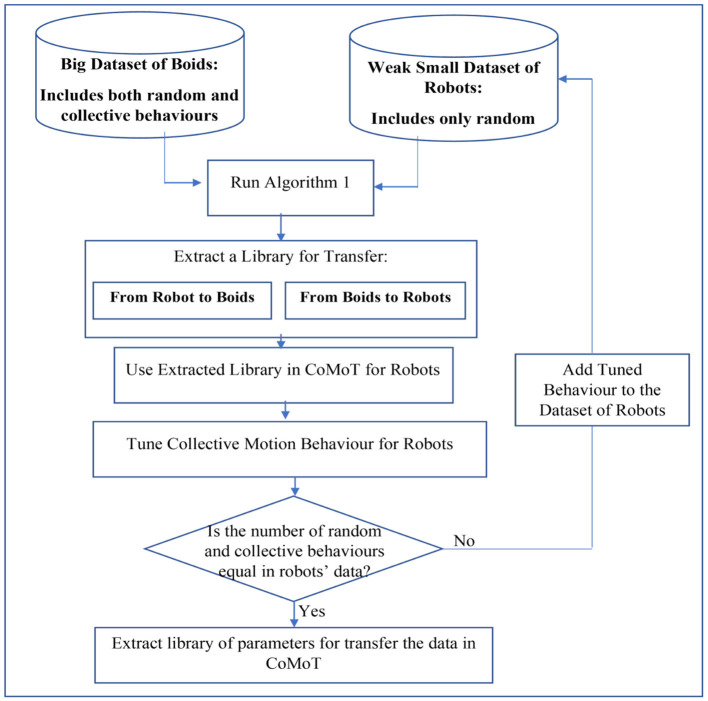
Iterative transfer learning to extract a library for CoMoT.

To overcome the need for hand-crafting collective behaviors in robots to create training data, an iterative approach is proposed in Figure 4. As shown in this figure, the robot data now includes only random behaviors. Similar to the one shot approach, the source and target data will be selected to generate libraries for transfer from boids to robots and inversely. Then the parameters for transfer from source to target data will be extracted. These parameters include the $\bar{S_{S^{\prime }}}$, $\bar{T_{j}}$, and the features of source data similar to the target data, corresponding to each feature *j* of the target data. The novel iterative approach is embedded with CoMoT to progressively enrich the library with collective behaviors. In each iteration the library of parameters for the transfer learning will be updated. The final library will be used in the observation space of CoMoT. Then, CoMoT uses this extracted library of parameters and Equation 3, to transfer the observation space of boids to robots. After transferring the observation space, CoMoT tunes a collective behavior for the updated data. Then by using the library of parameters to transfer from boids to robots, this tuned behavior will be transferred to robots. While the robot data is imbalanced (the number of random and collective behaviors are not the same), the automatically tuned behavior by CoMoT will be added to the robot data. Again by running Algorithm 1 in this updated data and the boid data, a new library of parameters will be generated. This will continue, iteratively, until the robot dataset becomes balanced with respect to the number of instances representing random and collective behaviors. This will enrich the library with both random and collective behaviors, with no need to hand-craft any collective behavior. The final library includes the parameter for transfer from boids to robots and conversely.

### 3.4. Automatic collective motion tuning for robots

**Figure 5:**
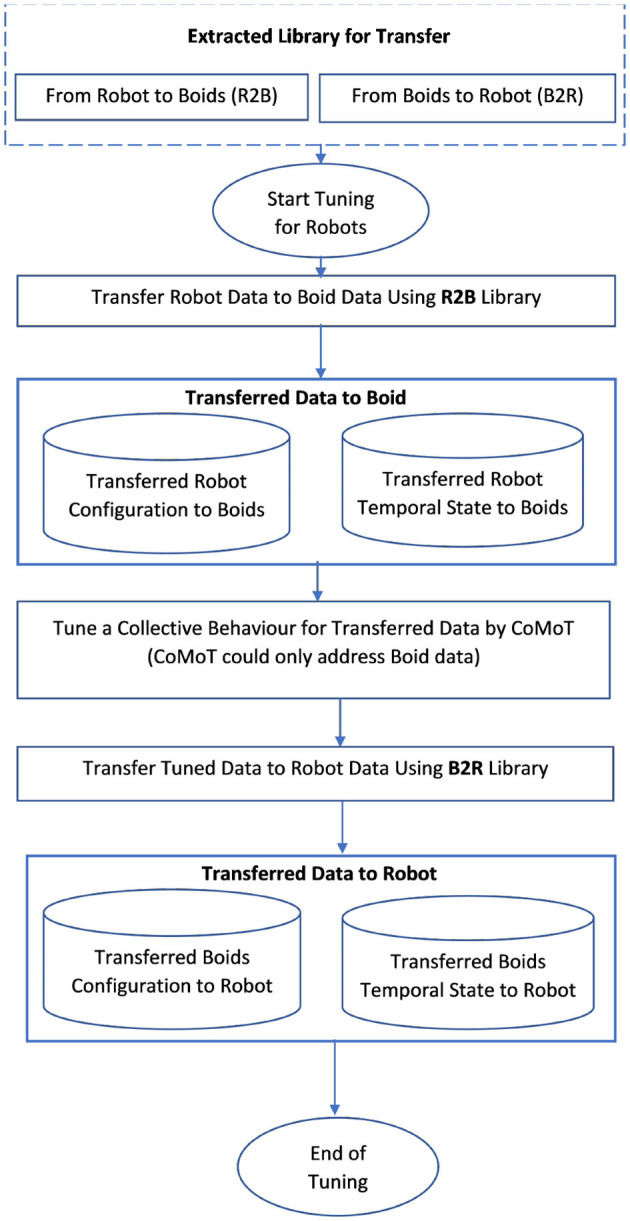
Transfer learning for collective motion tuning in robots.

Using the library of parameters extracted according to either Figure 3 or Figure 4, CoMoT is able to tune collective behavior for robots is <10 s comparing to Algorithm 1 which takes 10 min to provide the transfer learning parameters. The procedure works as shown in Figure 5. As mentioned earlier, the procedure of Algorithm 1 can be used in two ways: namely, it can be run considering the robot as the source and boids as the target data; AND it can be run considering boids as source and robot data as the target. In the first case, the extracted library from Algorithm 1 uses robots as the source and boids as the target and transfers the robot data to boids. Based on this transfer, the robot observation space will have a distribution and range similar to boids (both for configuration and temporal state parameters). Therefore, the observation space of this transferred data could be processed by CoMoT. However, CoMoT could only address boids data, and using this transfer, enables CoMoT to work with robot data. This is called an R2B library in Figure 5. Then CoMoT generates and performs the trial and error approach on the updated data and automatically tunes a collective behavior for the updated (transferred) data. Once, collective behavior is generated, in the second case, it can translate a tuned configuration output by CoMoT back to a distribution that will be feasible on the real robot platform. This is called the B2R library in Figure 5. As a result, a collective behavior will be generated for robots without designing RL from scratch or requiring extensive data collection.

## 4. Experiments and discussions

This section includes some experiments to evaluate the one shot and iterative approaches. The first subsection discusses the experimental setup and the scenarios used in the other subsections. The experiments in these subsections investigate the following aspects:

- The impact of transfer learning on the distribution and ranges of robots' observation space. In this experiment, boids data is the source problem, and robot data is the target problem.
- The quality of reward and formation of collective behaviors for robots tuned by CoMoT using both the one shot and iterative approaches.
- The ability of tuned behaviors to be used in coverage tasks, noting that CoMoT has not been trained for coverage.

The scenarios and experiments will be discussed in detail in the following sections.

### 4.1. Experimental setups

In the following experiments, two robot platforms are used: (1) the Simulated Pioneer 3DX robots in CoppeliaSim and (2) real Sphero BOLT robots. We created six random movement scenarios for each of these robot platforms, shown in Table 1. These six scenarios cover a diverse range of values for swarm parameters of cohesion, alignment and separation weights and their corresponding radii. These parameters are described in Section 2.1. The six scenarios for Pioneer 3DX robots are from Abpeikar et al. (2022a) while the Sphero BOLT scenarios were designed for this current paper.

**Table 1:** Parameters for random behaviors of Pioneer 3DX robots (Abpeikar et al., 2022a) and Sphero BOLT robots.

**Parameters**	***W*_*a*_**	***W*_*c*_**	***W*_*s*_**	***R*_*a*_**	***R*_*c*_**	***R*_*s*_**
**Pioneer 3DX random (PR)**						
PR1	1.00	0.00	1.20	2.00	2.00	1.00
PR2	1.20	0.10	1.20	2.00	2.00	1.00
PR3	1.00	0.50	1.20	2.00	2.00	1.00
PR4	0.05	0.05	0.90	2.00	2.00	1.00
PR5	0.01	0.20	1.00	2.00	2.00	1.00
PR6	0.05	0.25	0.90	2.00	2.00	1.00
**Sphero BOLT random (SR)**						
SR1	0.01	0.01	0.01	100.00	100.00	25.00
SR2	0.00	0.01	0.00	100.00	100.00	25.00
SR3	0.01	1.00	1.00	50.00	100.00	25.00
SR4	0.02	0.02	0.01	50.00	100.00	25.00
SR5	0.01	0.01	1.00	50.00	100.00	25.00
SR6	0.01	1.00	0.01	50.00	100.00	25.00

As mentioned in Section 3.3 for the one shot approach to library extraction there are also some hand-crafted, structured collective motion behaviors. These hand-crafted behaviors are defined in Table 2. The parameters were chosen following the guidelines of Khan et al. (2020). The hand-crafted behaviors for Pioneer 3DX robots are derived from Abpeikar et al. (2022a).

**Table 2:** Parameters for hand-crafted collective behaviors of Pioneer 3DX robots (Abpeikar et al., 2022a) and Sphero BOLT robots.

**Parameters**	***W*_*a*_**	***W*_*c*_**	***W*_*s*_**	***R*_*a*_**	***R*_*c*_**	***R*_*s*_**
**Pioneer 3DX collective behavior (PC)**						
PC1	1.00	0.10	1.20	2.00	2.00	1.00
PC2	1.00	0.10	1.50	2.00	2.00	1.00
PC3	1.00	0.05	1.50	2.00	2.00	1.00
PC4	1.20	0.05	1.50	2.00	2.00	1.50
PC5	1.20	0.05	1.50	2.00	2.00	1.00
PC6	1.20	0.05	1.50	2.00	2.00	1.00
**Sphero BOLT collective behavior (SC)**						
SC1	1.00	1.00	0.00	50.00	100.00	25.00
SC2	1.00	1.00	0.01	50.00	100.00	25.00
SC3	1.50	1.55	0.01	50.00	100.00	25.00
SC4	0.50	0.50	0.01	50.00	100.00	25.00
SC5	0.50	0.50	0.00	50.00	100.00	25.00
SC6	1.00	1.00	0.01	100.00	100.00	25.00

The simulation on Pioneer 3DX was selected since the scenarios were available from Kasmarik et al. (2020). As an example of real robots the Sphero BOLT robots are selected, as they permit us to run the short duration swarming experiments in this paper without need for an external positioning system. The Pioneer 3DX scenarios are run in a 20 × 20*m* simulated, walled environment in CoppeliaSim. The Sphero BOLT scenarios are run in a 2 × 2*m* real environment with virtual walls. These scenarios will be used in the following experiments.

### 4.2. Experiment 1: Impact of transfer learning on temporal state values of observation space

The aim of this experiment is to examine the impact of transfer learning on the distribution and ranges of target data observation space. This experiment uses boids data as the source data and Sphero BOLT data as the target data. It will investigate the changes in the distribution of Sphero BOLT data and how it could be mapped to the observation space of Boids. As discussed in Section 3.1, and in the work done by Abpeikar et al. (2022c), the observation space of CoMoT uses configuration parameters of *S* for a specific number of timesteps and extracts the temporal state values of all the agents during these timesteps. Then the knowledge base of CoMoT on these temporal state values could result in a reward or a penalty for the current state and action. Moreover, Algorithm 1 is designed to work with both the configuration parameters (*Data*_*conf*_) and temporal state parameters (*Data*_*temp*_). This experiment investigates the results of temporal state parameters since it has more features and more complex space.

**Figure 6:**
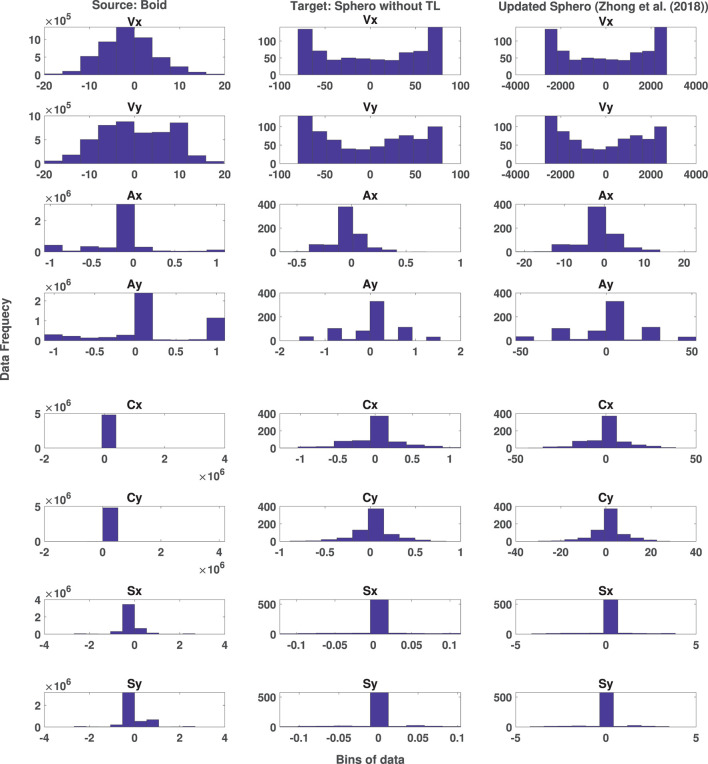
Distribution of Boid temporal state data (column 1) and original Sphero BOLT temporal state data (column 2) and the effect of transfer learning method proposed by Zhong et al. (2018) (column 3).

As has been discussed in Section 3.3.1, this paper proposed a transfer learning method, which is an extension of the transfer learning method of Zhong et al. (2018). Due to this extension, the new transfer learning method could prepare the observation space of robots to make them applicable and useful by CoMoT. The effect of the transfer learning proposed by Zhong et al. (2018) on the temporal state parameters of Sphero is presented in Figure 6. This figure shows the distribution and ranges of temporal parameters of source data (boids) in the first column and those of the target data (Sphero) in the second column. The effect of transfer learning of Zhong et al. (2018) on the target data is presented in the third column. As it is shown in this figure, there are many parameters, in which neither their distribution nor their ranges become similar to the distribution and ranges of the source data of boids. This indicates the need for designing a transfer learning applicable to the observation space of CoMoT. The results of this extension of transfer learning will be discussed in the next experiment.

This experiment investigates the effect of transfer learning on target data using both the one shot and iterative approaches. For the one shot approach, the Sphero BOLT data includes random behaviors (SR1-SR6) of Table 1 and collective behaviors (SC1-SC6) of Table 2. However, for the iterative approach, the data only includes random behaviors (SR1-SR6) of Table 1. Automatically generated collective behaviors will be added iteratively using CoMoT, as described in Section 3.3.2.

**Figure 7:**
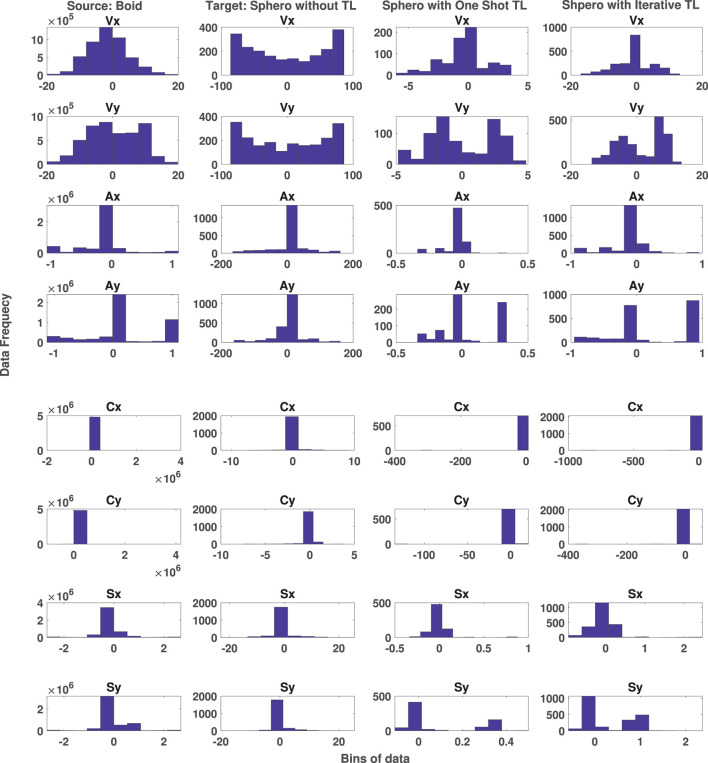
Distribution of Boid temporal state data (column 1) and original Sphero BOLT temporal state data (column 2) and the effect of one shot (column 3) and iterative (column 4) transfer learning approaches on distribution of original Sphero BOLT temporal state data.

The target data includes 713 records of temporal state values in *Data*_*temp*_. These 713 records are the outcomes of either the iterative approach or the one shot approach. This number of data records corresponds to approximately 30 s of total run-time for each scenario using the configuration parameters. This number of data records is very small compared to the 4,000,000 temporal state samples in the labeled boid data, which corresponds to approximately 1,500 timesteps (20 min) of total run-time for each scenario. It also took 6 months to collect this data from human participants. The number of features in both source and target data are the same, but with different ranges and distributions. The training processing time of Algorithm 1 takes about 10 min. Figure 7 shows the distribution of updated temporal state using the one shot and iterative approaches of transfer learning in columns 3 and 4, respectively. It also shows the distribution of the source data in column 1 and the target data in column 2 in the absence of transfer learning. Figure 7 includes the temporal state values of *x, y* coordinates of velocity *V*, alignment *A*, separation *S*, and cohesion *C*. As mentioned earlier the aim is to map the distribution of the target data of column 2 to the source data of column 1 using the one shot and iterative approach of transfer learning. As shown in this figure, not only the distribution of all the similar features of the target data without any transfer learning in column 2 is different to the source data of boids in column 1, but also the ranges are different. Applying the one shot transfer learning approach, we see in column 3 of Figure 7 that the distributions and the ranges in the target data are matched with the source data. Applying the iterative transfer learning approach, the distributions of updated target data in column 4 of Figure 7 and the source data are the same. In comparison to the one shot approach in column 3, the iterative approach in column 4, covers the larger part of the data ranges of the boid data. In conclusion, both one shot and iterative transfer learning methods could successfully map the temporal state of target data into the distribution and ranges of the temporal state of source data.

### 4.3. Experiment 2: Collective motion recognition

This experiment examines if the transferred target data in the frameworks of Figures 3, 4 can be correctly recognized as collective behavior or random (not-collective) behavior. As discussed in Section 3.3.2, applying the procedures of Figures 3, 4 using Algorithm 1 on a small dataset of robot behavior will generate a library for transferring between boids data and the robot data. In this experiment, the class labels of collective behaviors of Table 2 are set to 1 (*class* = 1). Also, the class labels of random (not-collective) behaviors of Table 1 are set to zero (*class* = 0). We then feed the data through the knowledge base of Section 3.1. For the one shot approach, the library of each robot is generated over all the corresponding behaviors of Tables 1, 2. For the iterative approach, the library includes the random behaviors of Table 1 and the automatically tuned collective behaviors that are iteratively added to the library.

Table 3, shows the accuracy, precision, and recall metric values of the knowledge base in collective behavior recognition of robots. As shown in this table, the updated Pioneer 3DX and Sphero BOLT data using extracted libraries could generate accurate machine-recognizable behaviors using both one shot and iterative approaches. However, using the iterative approach provides higher performance in comparison to the one shot approach. As a result, these libraries can accurately transfer any new observation of Sphero BOLT robots or Pioneer 3DX robots to boids to be used in CoMoT. Recently a transfer learning approach was proposed to enable the collective behavior recognition in simulated robots (Abpeikar et al., 2022b). However, despite the current paper, this transfer learning could only address the collective behavior recognition, and is not applicable in collective behavior tuning for robots.

**Table 3:** Performance of collective motion recognition on transferred Sphero and Pioneer 3DX data using Knowledge-base of Section 3.1.

**Data**	**One shot TL**			**Iterative TL**		
	**Accuracy (%)**	**Precision**	**Recall**	**Accuracy (%)**	**Precision**	**Recall**
Sphero BOLT	93.25 ±2.28	0.90 ±0.25	0.92 ±0.41	100.00 ±0.00	1.00 ±0.00	1.00 ±0.00
Pioneer 3DX	98.12 ±0.68	0.96 ±0.17	0.99 ±0.01	99.65 ±0.22	0.98 ±0.12	0.99 ±0.01

In addition, the transfer procedure for each new sample of the Pioneer 3DX robot, and Sphero BOLT robot is also investigated. Regarding this investigation the maximum transfer time for both the Pioneer 3DX robot, and Sphero BOLT robot is 10 s, using the extracted libraries.

As shown in Figure 2, the knowledge base of Section 3.1 is the reward signal generator for CoMoT. Therefore, being able to correctly recognize the behaviors using the extracted library, has the following advantages:

- There is no need to run Algorithm 1 multiple times during the tuning procedure. It can simply be run once offline and the generated library used henceforth. This will reduce the tuning time to <10 s.
- It indicates that the transferred data have the same characteristics as the original data, which makes them machine recognizable as collective or not-collective behavior. This will eliminate the need for expensive collection of human labeled data.
- It indicates that the reward signal of CoMoT is likely to generate the correct reward, and consequently tune accurate collective behaviors. This will be confirmed in the following section.

### 4.4. Experiment 3: Automatic collective motion tuning for robots

The aim of this experiment is to investigate the quality of tuned behaviors for robots using transfer learning on observation space of CoMoT. This experiment uses the library achieved from Figures 3, 4, and CoMoT with the transfer learning on the observation space (Figure 5), to automatically tune collective behaviors from the random behaviors of Table 1 for Pioneer 3DX and Sphero BOLT robots. To evaluate the tuned behaviors, the reward value of CoMoT with one shot and iterative transfer learning has been investigated. As mentioned in Abpeikar et al. (2022a,c), a cumulative reward signal in the range of [250, 500] indicates a likely collective behavior, while a higher reward indicates more accurate and reliable collective behavior.

**Figure 8:**
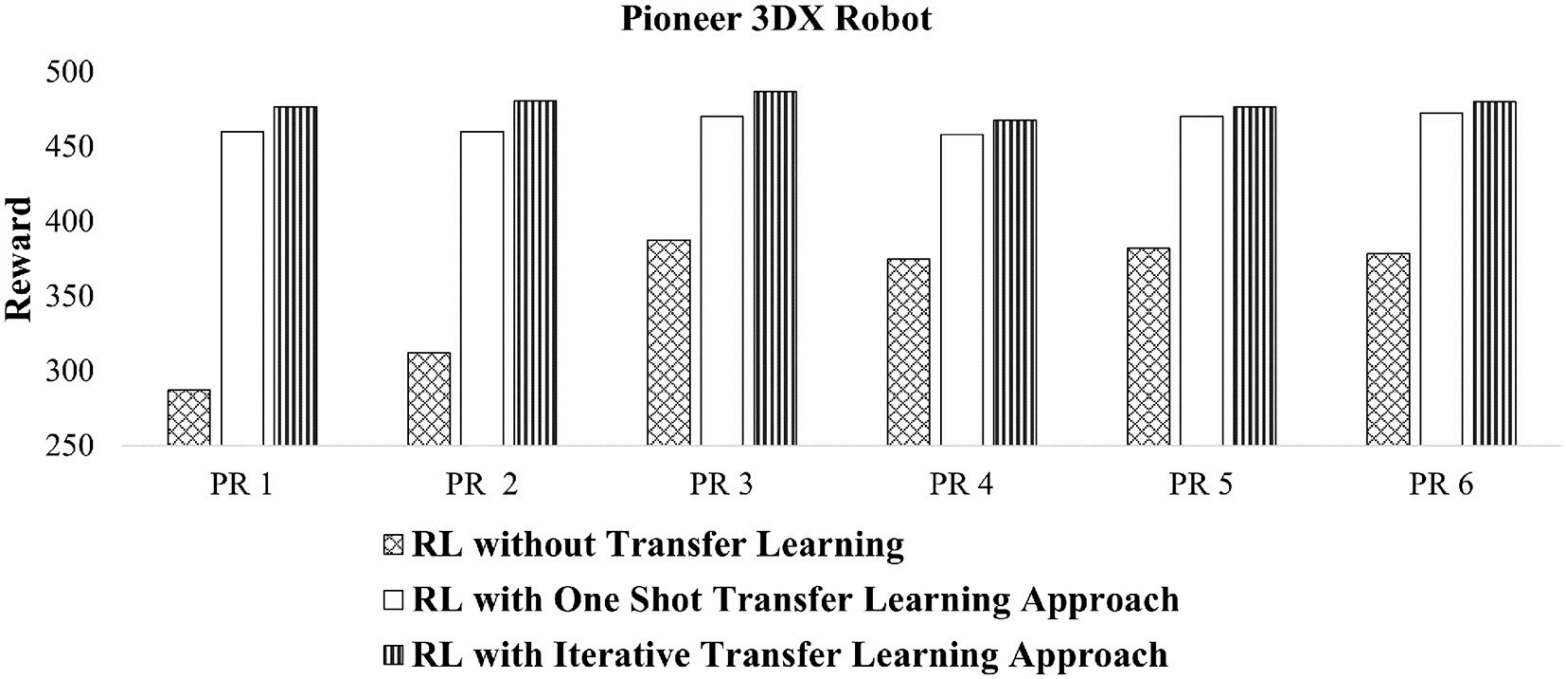
Reward values of tuned collective behaviors for Pioneer 3DX robots using CoMoT with transfer learning as in Figure 5.

For the Pioneer 3DX robots, we use the results from Abpeikar et al. (2022a,c) for the baseline “without transfer learning” case. Figure 8, compares the reward signal of CoMoT with the two approaches of transfer learning with that of Abpeikar et al. (2022a,c) without any transfer learning. This figure shows the reward signal for collective motion tuning of Pioneer 3DX robots increased when applying the transfer learning. The average reward values for the 6 scenarios without transfer learning, with one shot transfer learning, and with iterative transfer learning is 353.61 ± 31.30, 465.12 ± 4.64, and 478.16 ± 4.62, respectively. Moreover, it indicates that the reward values while applying the iterative transfer learning approach are higher than the one shot transfer learning approach.

In contrast, on Sphero BOLT robots the observation space range and the distribution are very different to point-mass boid data. Hence, they are not compatible with the range and distribution of the observation space that CoMoT has been trained on. Therefore, it is not possible to apply CoMoT to tune Sphero BOLT behavior without transfer learning. This indicates the significance of using transfer learning on CoMoT for real robots.

**Figure 9:**
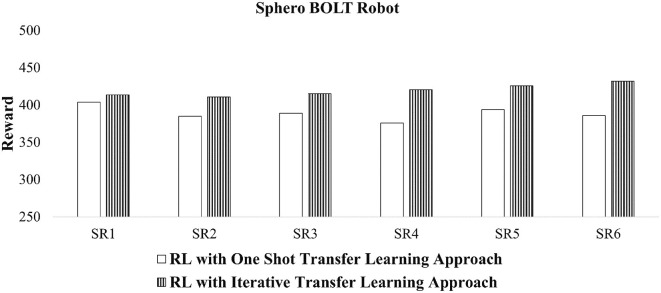
Reward values of tuned collective behaviors for Sphero BOLT robots using CoMoT with transfer learning as in Figure 5.

Figure 9 shows that when transfer learning is applied, suitable reward values are returned. In this figure, all the cumulative reward signals are >250, which indicates that the tuned behaviors are likely to be collective behaviors. The average reward values of these six scenarios with one shot transfer learning is 389 ± 6.88. This average value for the iterative transfer learning is 419.75 ± 5.91. In addition to this, the iterative transfer learning approach provides higher reward values than the one shot transfer learning, consistent with earlier experiments.

**Figure 10:**
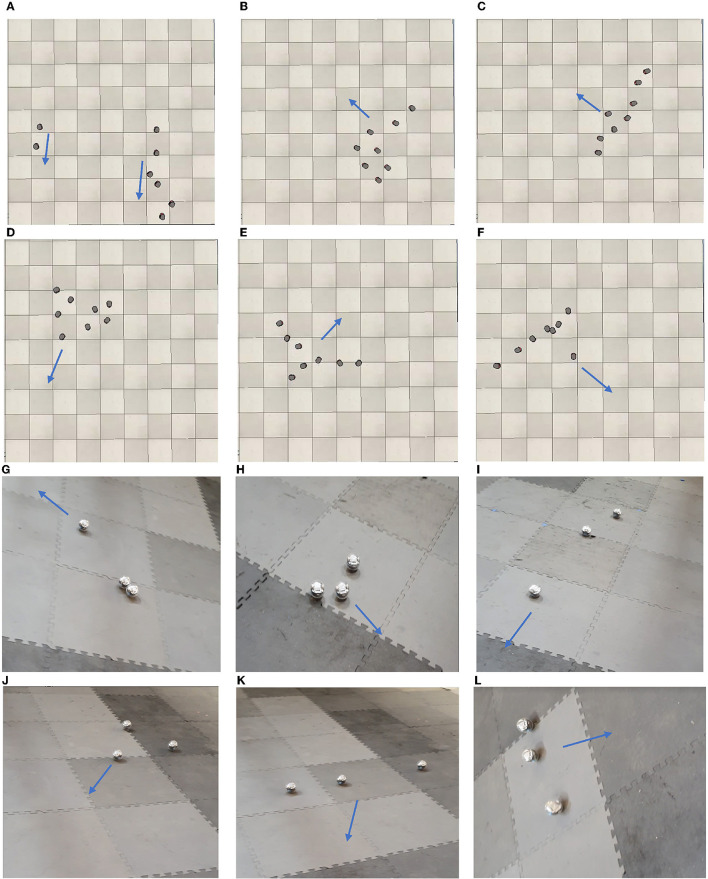
Formation of automatic tuned behaviors in robots using CoMoT with iterative transfer learning approach (The robots keep the same formation during the movement and the blue arrow shows the direction of movement). **(A)** Tuned behavior for PR1. **(B)** Tuned behavior for PR2. **(C)** Tuned behavior for PR3. **(D)** Tuned behavior for PR4. **(E)** Tuned behavior for PR5. **(F)** Tuned behavior for PR6. **(G)** Tuned behavior for SR1. **(H)** Tuned behavior for SR2. **(I)** Tuned behavior for SR3. **(J)** Tuned behavior for SR4. **(K)** Tuned behavior for SR5. **(L)** Tuned behavior for SR6.

**Figure 11:**
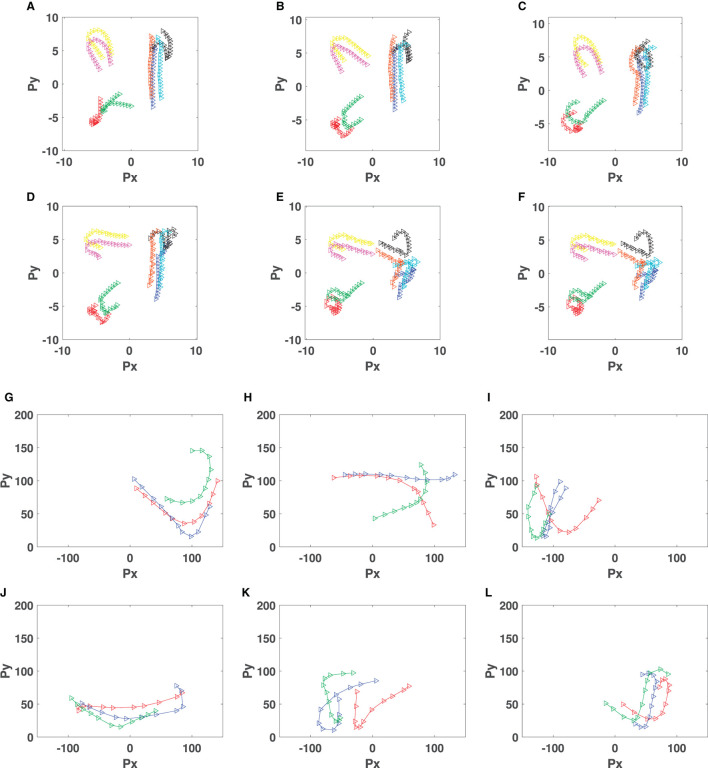
Formation of automatic tuned behaviors in robots using CoMoT with iterative transfer learning approach (The robots keep the same formation during the movement and the blue arrow shows the direction of movement). **(A)** Tuned behavior for PR1. **(B)** Tuned behavior for PR2. **(C)** Tuned behavior for PR3. **(D)** Tuned behavior for PR4. **(E)** Tuned behavior for PR5. **(F)** Tuned behavior for PR6. **(G)** Tuned behavior for SR1. **(H)** Tuned behavior for SR2. **(I)** Tuned behavior for SR3. **(J)** Tuned behavior for SR4. **(K)** Tuned behavior for SR5. **(L)** Tuned behavior for SR6.

Figure 10, shows some snapshots of the formations and movement directions of tuned behaviors for Pioneer 3DX robots (Figures 10E, F) and Sphero BOLT robots (Figures 10G, L). For Pioneer 3DX we examined tuned behaviors with eight robots. For the Sphero BOLTS we examined tuned behaviors with three robots, the maximum we can reliably control with a single Bluetooth connection. As shown in Figure 10, the Pioneer 3DX and Sphero BOLT robots move close to each other, and in the same direction.^3^ The trajectories of robots generating the collective motion behaviors of Figure 10 is presented in Figure 11. In this figure, the trajectory of eight Pioneer3DX robots is presented for 1,000 timesteps (since they moving slowly). The trajectory of three Sphero robots is presented for 10 timesteps (since they move faster). Due to the fast movement of Sphero BOLT robots in some cases, they collide with each other, but there is no distraction in their movement, and they keep the same formation. Colliding between Sphero BOLT robots is not an issue, due to their design with a plastic shell, which makes them resistant to damage while colliding with any object. Moreover, the positioning and magnetometer sensors of Sphero are prone to inaccuracies. The positioning is done using odometry from the wheel encoders and is subject to drift. Therefore, the simulations are run for 2 min before drifting happens. The accuracy of the magnetometer heading sensor is set to 360°, which is from 0° to 180° to left and from 0° to −180° to right. The inaccuracies will also affect the computations of cohesion, separation, and alignment. On the other hand, as mentioned in Section 3.1 the knowledge base of CoMoT uses if-then rules to make decisions based on the behavior of all of the robots moving in a specific period of time, not an individual robot in one timestep. Therefore, this aggregation of the robots' motion helps in managing these inaccuracies in the collective behavior-tuning procedure of CoMoT. However, running the simulation for longer than 2 min, and using more Sphero robots in a bigger environment might result in higher inaccuracies, because of the more drifting which might happen in the movement of the groups of robots. This could be an avenue for future work.

In addition to the improvements discussed above, CoMoT requires 2,000 episodes, each with a maximum of 100 steps. Using transfer learning eliminates this training procedure and collective motion tuning for robots is done in one episode and in <10 steps. The training data for CoMoT was collected over a period of 6 months. Using transfer learning, a small set of training data for robots could be collected in just 1 day.

### 4.5. Experiment 4: Coverage ability of tuned behaviors

The aim of this experiment is to demonstrate an application of tuned behaviors. We examine a coverage problem in this section. Multi-robot coverage is an interesting problem with different possible approaches. One class of approach assumes each robot will cover an independent region of the environment. More recently, there has been interest in swarming approaches where robots stay together so that there is redundancy if some robots fail (Tran et al., 2022).

This section investigates the coverage performance of the tuned collective behaviors of Figure 10 compared to the untuned behaviors (behaviors of Table 1). Each behavior of the Sphero BOLT robots was run in a 2 × 2*m* rectangular area sorrounded with virtual walls. The behaviors of the Pioneer 3DX robots were run in the simulated 20 × 20*m* rectangular walled area. These areas were both considered using a 10 × 10 grid cell environment for coverage purposes. A grid cell is covered if at least one robot visits that grid cell. Table 4 shows the coverage percentage of each of the tuned behaviors of Figure 10, and the maximum time required to achieve this coverage. As mentioned in this table, the maximum coverage of the tuned behaviors in Sphero robots is more than 88% of the area, in <0.26 min (16 s). Also, the maximum coverage of tuned behaviors in Pioneer 3DX robots is more than 86% of the area in <5 min.

**Table 4:** Coverage performance of tuned collective behaviors from Figure 10.

**Behavior**	**Coverage (%)**	**Time (min)**
**Pioneer 3DX**		
PR1	92.00	3.91
PR2	100.00	3.21
PR3	92.00	4.30
PR4	86.00	3.21
PR5	90.00	4.50
PR6	96.00	4.54
**Sphero BOLT**		
SR1	92.00	0.25
SR2	100.00	0.26
SR3	100.00	0.22
SR4	88.00	0.26
SR5	97.00	0.24
SR6	97.00	0.26

**Figure 12:**
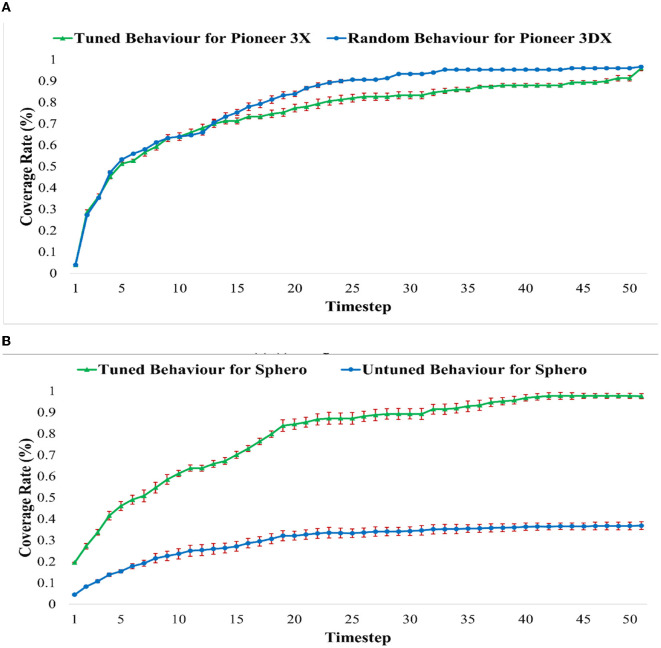
The coverage rate over time for the tuned behaviors of Figure 10 and random behaviors of Table 1. **(A)** Average coverage rate of untuned behavior of PR1–PR6 of Table 1 and tuned behaviors of motions (A–F) of Figure 10 for Pioneer3DX Robots. **(B)** Average coverage rate of untuned behavior of SR1–SR6 of Table 1 and tuned behaviors of motions (G–L) of Figure 10 for Sphero Robots.

The changes in the coverage rate over time for each of the behaviors including the initial untuned behaviors of Table 1 and the tuned behaviors of Figure 10, are presented in Figure 12. We can see that the tuned behaviors achieve coverage no more slowly than the untuned behaviors with the added benefit that the tuning keeps the robots swarming together.

## 5. Conclusion and future work

Automatically tuning a collective behavior for robots is important, since it is time-consuming for a human to manually tune behaviors for every new robot platform they wish to use for swarming systems. The problem becomes harder if the swarm includes different types of robots. Recent works developed an RL approach called CoMoT for automatically tuning collective behavior for boids and simulated robots. However, designing such a method on real robots is costly in terms of data collection, and the risk of a trial-and-error tuning procedure. This paper has shown that it is possible to transfer knowledge of CoMoT from point mass data to real robots, requiring only a small amount of additional data.

One shot and iterative transfer learning approaches were used to extract a library which transfers data from boids to robots and inversely. The one shot approach uses some small set of hand-crafted random and tuned behaviors to generate the transfer library. The iterative approach only uses a set of random data, and iteratively adds collective behavior to the library by recursively calling CoMoT. The extracted library then is used in CoMoT to transfer the observation space of robots to boids.

After applying CoMoT reinforcement learning on this transferred data and automatically tuning a collective behavior, the data then will transfer back to the robots. This will result in a fast (<10 s) automatic collective behavior tuning for robots without any further training required.

The robot platforms used in this paper are the Pioneer 3DX and Sphero BOLT robots. The tuned behaviors could provide some known formations of flocking and line behavior in robots. Moreover, although the CoMoT has not been trained to provide coverage, the tuned behaviors using transfer learning could produce a good coverage performance. Despite these achievements, there are some challenges which open an avenue for future works, as follows:

- First, we would like to investigate whether it is possible to re-use a transferred dataset to permit CoMoT to tune behaviors on a different robot platform with similar feature distribution, without collecting further data. This would mean, for example, that a swarm of robots could themselves teach a new member how to behave within their swarming system.
- Second, this paper investigates the use of transfer learning on observation space of reinforcement learning. However, another approach for collective motion tuning is to use evolutionary approach rather than reinforcement learning. Investigating the performance of transfer learning on evolutionary approach to switch between the robot platforms, could be an avenue for future studies.
- Another area of future work lies in generating greater diversity in tuned behaviors. Our current system has shown promise for generating grouped and aligned movement of robots. However, other collective motions such as dispersion or movement in a line are also possible. Recognizing and tuning such behaviors remains an area for future work.

## Data availability statement

Publicly available datasets were analyzed in this study. This data can be found at: https://dataverse.harvard.edu/dataset.xhtml?persistentId=doi:10.7910/DVN/S1YJOX.

## Author contributions

The main idea of transfer learning has been proposed and also, the algorithm design and experiments are done and paper has been written by SA. The python code for Sphero BOLT robots is written by KK. SA and KK were responsible for data collection. Proofreading and checking the outcomes have been done by KK and MG. All authors contributed to the article and approved the submitted version.

## Footnotes

^1^ https://support.sphero.com/article/c11rxl3nsf-what-are-sphero-bolt-s-live-sensors

^2^A PDF version of the screenshots captured from this online survey is downloadable from the following link, by simply clicking the message shown on the page: https://unsw-swarm-survey.netlify.app/.

^3^The videos of the collective behavior motions captured in UNSW Robotic Lab are available online on YouTube: https://www.youtube.com/playlist?list=PL3FDKnREgAf-bmVhm1W6YTRnhHgm-iEis.
